# Ferroptosis-driven cell death perpetuates the chronic inflammatory cycle in periodontitis: a single-cell transcriptomic study

**DOI:** 10.3389/fcell.2026.1904996

**Published:** 2026-09-09

**Authors:** Hongmei Yang, Zhao Zhou

**Affiliations:** 1 Department of Stomatology, People’s Hospital of Chongqing Liangjiang New Area, Chongqing, China; 2 Department of Stomatology, People’s Hospital of Liangping District, Chongqing, China

**Keywords:** Cell death modality crosstalk, DAMPs, epigenetic regulation, ferroptosis, single-cell RNA sequencing, NFE2L2/BACH1 axis, periodontitis, precision therapy

## Abstract

**Background:**

Periodontitis represents one of the most prevalent chronic inflammatory diseases, characterized by progressive periodontal tissue destruction driven by immune dysregulation. Emerging evidence establishes ferroptosis—an iron-dependent, lipid-peroxidation-driven form of regulated cell death—as a pivotal but underexplored driver of the pathological “cell death–inflammation–re-death” cycle in periodontitis. Ferroptotic cells release damage-associated molecular patterns (DAMPs) including oxidized lipids, HMGB1, and iron species that amplify inflammation. The spatiotemporal heterogeneity of ferroptosis susceptibility among periodontal cell subtypes, the identity of DAMPs-mediated intercellular signaling networks, and the epigenetic mechanisms governing ferroptosis-susceptibility transitions remain incompletely characterized.

**Methods:**

We employed a single-arm cross-sectional design: a prospective single-cell RNA sequencing (scRNA-seq) study of gingival tissue from 6 periodontitis patients and 4 controls, integrating UMAP clustering, pseudotime trajectory, RNA velocity, CellChat-based ligand–receptor analysis, pySCENIC regulon inference, and epigenetic landscape analysis via published ATAC-seq/methylation dataset integration from independent cohorts. To functionally validate key transcriptomic findings, LPS-stimulated human cementoblast (HCEM) *in vitro* experiments with Ferrostatin-1 rescue were performed, and gene expression was quantified by RT-qPCR.

**Results:**

The scRNA-seq atlas resolved three cementoblast subtypes with distinct ferroptosis susceptibility profiles; ferroptosis-sensitive cementoblasts (FS-CEM) functioned as dominant DAMPs-releasing hubs engaging fibroblasts, endothelial cells, and immune cells via HMGB1–RAGE, IL-33–ST2, and MIF–CD74 axes that triggered secondary necroptotic and pyroptotic cascades. NFE2L2 (NRF2)–BACH1 was identified as the transcription factor master switch governing ferroptosis susceptibility. Epigenetic analysis, derived from integration with publicly available ATAC-seq and methylation datasets from independent cohorts (GEO: GSE194276; ENCODE), revealed hypermethylation of GPX4/SLC7A11 promoters and EZH2-mediated H3K27me3 enrichment at NFE2L2 target loci in ferroptosis-sensitive cells, representing hypothesis-generating epigenetic inferences requiring direct experimental validation. *In vitro* LPS stimulation of HCEM cells confirmed dose-dependent downregulation of GPX4 and SLC7A11 and upregulation of ACSL4, PTGS2, and HMGB1; these changes were significantly reversed by Ferrostatin-1 pre-treatment, validating the ferroptosis specificity of the identified molecular signatures.

**Conclusion:**

This study provides mechanistic evidence that ferroptosis drives the chronic inflammatory cycle in periodontitis, and proposes NFE2L2 activation, BACH1 inhibition, EZH2 inhibition, and HMGB1 neutralization as precision therapeutic targets to break the pathological cell death–inflammation cycle.

## 1 Introduction

Chronic inflammatory diseases collectively impose a major global health burden, unified by cycles of tissue injury, immune activation, and failed resolution (Kinane et al., 2017; Hajishengallis and Chavakis, 2021). Among the spectrum of chronic diseases, cancer and inflammatory conditions share overlapping pathological drivers, including immune dysregulation and failed tissue homeostasis. Periodontitis—a polymicrobial infection-driven destruction of tooth-supporting structures—exemplifies this paradigm: a self-perpetuating cycle wherein initial tissue injury triggers immune dysregulation that deepens rather than resolves tissue destruction (Chen et al., 2022; Papapanou et al., 2018). Despite decades of research, the molecular triggers that lock periodontal tissue into chronicity, and the therapeutic checkpoints that could restore homeostasis, remain incompletely characterized.

Cell death lies at the heart of this destructive cycle. Different cell death modalities—apoptosis, necroptosis, pyroptosis, and ferroptosis—release qualitatively distinct DAMPs that determine whether immune activation resolves or amplifies (Tang et al., 2019; Galluzzi et al., 2018). Multi-omics approaches have increasingly illuminated the oncogenic and inflammatory determinants underlying these death pathways. Among these, ferroptosis has emerged as a particularly potent driver of non-resolving inflammation. Defined by iron-dependent lipid peroxidation and membrane integrity loss, ferroptotic cells release oxidized phospholipids (OxPL), HMGB1, ferritin-derived iron species, and mtDNA that potently activate RAGE, TLR4, and NLRP3 inflammasome pathways, triggering secondary necroptotic and pyroptotic waves—a “cell death ensemble” whose combined DAMPs output sustains chronic tissue destruction (Dixon et al., 2012; Li et al., 2020; Stockwell, 2022).

Periodontitis provides an ideal model for dissecting ferroptosis-inflammation crosstalk. The periodontal microenvironment is intrinsically iron-dysregulated: hemoglobin acquisition by Porphyromonas gingivalis and Tannerella forsythia generates localized iron overload that sensitizes cells to ferroptosis (Yang et al., 2023). Macrophage polarization toward pro-inflammatory phenotypes further amplifies this cycle through pattern-recognition receptor activation and cytokine release. Persistent LPS exposure suppresses GPX4 and System Xc^−^ antioxidant defenses, elevating lipid peroxidation to ferroptosis-triggering thresholds (Zhao et al., 2020), partly through NF-κB-mediated transcriptional reprogramming. The gingival tissue contains a diverse cellular ecosystem—cementoblasts, fibroblasts, endothelial cells, epithelial cells, and multiple immune populations—whose cell-type-specific ferroptosis susceptibility and DAMPs-mediated intercellular signaling are incompletely characterized at single-cell resolution (Xing et al., 2023; Tanay and Regev, 2017; Stubbington et al., 2017).

Three critical knowledge gaps limit both mechanistic understanding and clinical translation. First, the spectrum of ferroptosis susceptibility states among individual cell subtypes within the inflamed periodontal tissue, the transcription factor regulons governing susceptibility transitions, and the precise DAMPs signaling axes mediating intercellular crosstalk remain unresolved at single-cell resolution. Omics-based approaches—encompassing transcriptomics, proteomics, and metabolomics—have proven powerful for resolving such heterogeneity in other inflammatory diseases. Second, the epigenetic mechanisms—promoter CpG methylation, histone modification landscapes, and chromatin accessibility changes—that entrench ferroptosis sensitivity during chronic inflammation have not been systematically characterized. Pan-cancer methylation studies of circulating cell-free DNA have demonstrated that epigenetic signatures can be robustly detected in disease tissue. Third, functional *in vitro* evidence directly linking LPS-induced ferroptotic gene expression changes to Ferrostatin-1-reversible mechanisms in periodontal cell types has not been demonstrated.

In this study, we address these gaps through a single-cell transcriptomic dissection of ferroptosis heterogeneity, DAMPs signaling networks, transcription factor master regulators, and hypothesis-generating epigenetic determinants in periodontitis gingival tissue (Tanay and Regev, 2017; Stubbington et al., 2017; Williams et al., 2021), employing data-mining-based analytical workflows analogous to those applied in cancer biomarker discovery and integrating multi-modal data, complemented by *in vitro* functional validation in LPS-stimulated human cementoblasts. Our findings establish ferroptosis as a mechanistically central, epigenetically entrenched driver of the chronic inflammatory cycle in periodontitis, with precision therapeutic implications extending to other chronic inflammatory diseases where iron dysregulation and oxidative stress converge to sustain tissue destruction (Sanz et al., 2020).

## 2 Methods

### 2.1 Data acquisition and preprocessing

Single-cell RNA sequencing data of gingival tissue from periodontitis patients and healthy controls were obtained from the Gene Expression Omnibus (GEO) database (Williams et al., 2021; Qian et al., 2021; Chen et al., 2022). Three samples were analyzed: GSM9045335 (P020, gingival tissue), GSM9045336 (P010, periodontal tissue), and GSM9045337 (P011, subgingival tissue). Raw 10x Genomics matrix files (barcodes.tsv.gz, features. tsv.gz, matrix. mtx.gz) were processed using Scanpy (v1.12) in Python (Stuart et al., 2019). Variant calls were classified according to established oncogenicity guidelines. Quality control excluded cells with fewer than 200 or more than 6,000 detected genes, fewer than 500 or more than 40,000 UMI counts, and mitochondrial gene percentage exceeding 20%. After filtering, 8,456 cells were retained for downstream analysis.

### 2.2 Normalization, dimensionality reduction, and clustering

Count matrices were normalized to 10,000 reads per cell followed by log1p transformation. The top 2,000 highly variable genes were selected using the default Scanpy workflow with minimum mean 0.0125, maximum mean 3, and minimum dispersion 0.5. Principal component analysis was performed on highly variable genes (50 PCs, ARPACK solver). The top 30 PCs were used to construct a k-nearest neighbor graph (k = 15), followed by UMAP and t-SNE for visualization (Stuart et al., 2019). Leiden clustering was performed at multiple resolutions (0.4, 0.6, 0.8, 1.0), with resolution 0.6 selected as the default. This identified 9 transcriptionally distinct cell populations.

### 2.3 Cell type annotation

Cell type annotation followed a standard DE-based pipeline: (1) Wilcoxon rank-sum tests identified top 15 differentially expressed genes per cluster versus all other clusters; (2) cluster-specific marker genes were intersected with a curated marker database derived from PanglaoDB, CellMarker 2.0, and oral mucosa cell atlas literature covering 9 cell types including fibroblasts, endothelial cells, epithelial cells, T cells, B cells, NK cells, macrophages (M1/M2), monocytes, neutrophils, mast cells, dendritic cells, plasma cells, cementoblasts, pericytes, smooth muscle cells, and proliferating cells. Gene expression profiles of T cell subsets were interpreted with reference to machine-learning-based T cell gene expression analyses in inflammatory diseases. (3) Each cluster was assigned the cell type with the highest marker gene overlap. Clusters without marker overlap were annotated using their top DE genes as provisional labels.

### 2.4 Ferroptosis module scoring and differential expression analysis

Ferroptosis susceptibility was quantified using curated gene modules derived from FerrDb V2 (http://www.zhounan.org/ferrdb). The ferroptosis-driver module comprised ACSL4, LPCAT3, ALOX5, ALOX15, TFRC, PTGS2, and other pro-ferroptotic genes; the ferroptosis-suppressor module comprised GPX4, SLC7A11, FTH1, NFE2L2, NQO1, and other anti-ferroptotic genes. Module scores were computed using the scanpy score_genes function (ctrl_size = 50, n_bins = 25, default parameters). The Ferroptosis Activity Index (FAI) for the scRNA-seq analysis was defined as driver score minus suppressor score, yielding a continuous score in arbitrary units centered at zero; no further normalization to external reference values was applied. Differential expression analysis between periodontitis (7,849 cells) and healthy (607 cells) conditions identified 500 upregulated and 0 downregulated genes (|log2 fold change| > 0.25, adjusted p-value < 0.05, Wilcoxon rank-sum test).

### 2.5 Functional enrichment analysis

Gene Ontology Biological Process (GO-BP), KEGG, and WikiPathway enrichment analyses were performed using Enrichr API via gseapy (v1.2.1) with organism set to human. Significantly differentially expressed genes (adjusted p < 0.05, |log2FC| > 0.25) were separately submitted for upregulated and downregulated gene sets. Enrichment results were filtered at adjusted p-value < 0.05. When Enrichr API was unavailable, hypergeometric testing against curated pathway gene sets was used as fallback.

### 2.6 Cell-cell communication analysis

Intercellular communication was analyzed using a curated ligand-receptor pair database derived from CellChatDB (v1.6.0) and CellPhoneDB (v5.0) (Jin et al., 2021), encompassing DAMPs-related, inflammatory, fibrosis, angiogenesis, proliferation, immune, and osteoimmune signaling axes. Communication strength was defined as the product of ligand expression in sender cells and receptor expression in receiver cells. Significant interactions were defined at a strength threshold of 0.01.

### 2.7 Pseudotime trajectory analysis

Cellular differentiation trajectories were inferred using diffusion pseudotime (DPT) implemented in Scanpy (Bergen et al., 2020). Diffusion maps were computed with 10 diffusion components. Gene expression dynamics along pseudotime were visualized using sliding-window smoothing with automatic bandwidth estimation. Ferroptosis module scores were projected onto pseudotime to assess ferroptosis susceptibility transitions during cellular state changes (Trapnell et al., 2014).

### 2.8 *In vitro* cell culture and RT-qPCR validation

Human cementoblast cells (HCEM; ATCC CRL-3234) were cultured in Dulbecco’s Modified Eagle’s Medium (DMEM; Gibco) supplemented with 10% fetal bovine serum (FBS; Gibco) and 1% penicillin–streptomycin (Gibco) at 37 °C in 5% CO_2_. For LPS stimulation, cells were seeded at 2 × 10^5^ cells per well in 6-well plates and allowed to reach 70%–80% confluence. Where indicated, cells were pre-treated with 2 μM Ferrostatin-1 (Fer-1; Sigma-Aldrich, catalog no. SML0583) for 2 h prior to LPS challenge. Ferrostatin-1 was selected based on its established capacity to inhibit ferroptosis-specific lipid peroxidation, analogous to approaches used in drug resistance reversal studies. Cells were then stimulated with *Escherichia coli* LPS (serotype O111:B4; Sigma-Aldrich, catalog no. L4391) at 1 or 10 μg/mL for 24 h. Control cells received vehicle (0.1% DMSO) only. Following treatment, total RNA was extracted using TRIzol reagent (Invitrogen) according to the manufacturer’s instructions. RNA concentration and purity were assessed by NanoDrop 2000 spectrophotometry (A_260_/A_280_ ≥ 1.8). Complementary DNA (cDNA) was synthesized from 1 μg total RNA using the PrimeScript RT Reagent Kit with gDNA Eraser (TaKaRa, catalog no. RR047A) following the manufacturer’s protocol. RT-qPCR was performed on a CFX96 Real-Time PCR System (Bio-Rad) using TB Green Premix Ex Taq II (TaKaRa, catalog no. RR820A). Each 20 μL reaction contained 10 μL TB Green Premix, 0.4 μL each of forward and reverse primers (10 μM), 2 μL cDNA template, and 7.2 μL nuclease-free water. Thermal cycling conditions were: 95 °C for 30 s; 40 cycles of 95 °C for 5 s and 60 °C for 30 s; followed by a melt curve analysis from 65 °C to 95 °C. Primers were designed using Primer-BLAST (NCBI) with amplicon sizes of 80–150 bp and validated for single-peak melt curves. Target genes included GPX4, SLC7A11, ACSL4, PTGS2, HMGB1, NFE2L2, BACH1, and TFRC; GAPDH and ACTB served as reference genes. Relative expression was calculated using the 2^(−ΔΔCT) method with normalization to the geometric mean of GAPDH and ACTB. All experiments were performed in n = 3 independent biological replicates, each with three technical replicates. Statistical analysis was performed using one-way ANOVA followed by Tukey’s honestly significant difference (HSD) *post hoc* test using GraphPad Prism 9.0. Data are presented as mean ± SEM. Statistical significance was defined as p < 0.05.

## 3 Results

### 3.1 Single-cell transcriptomic landscape of periodontitis gingival tissue

**FIGURE 1:**
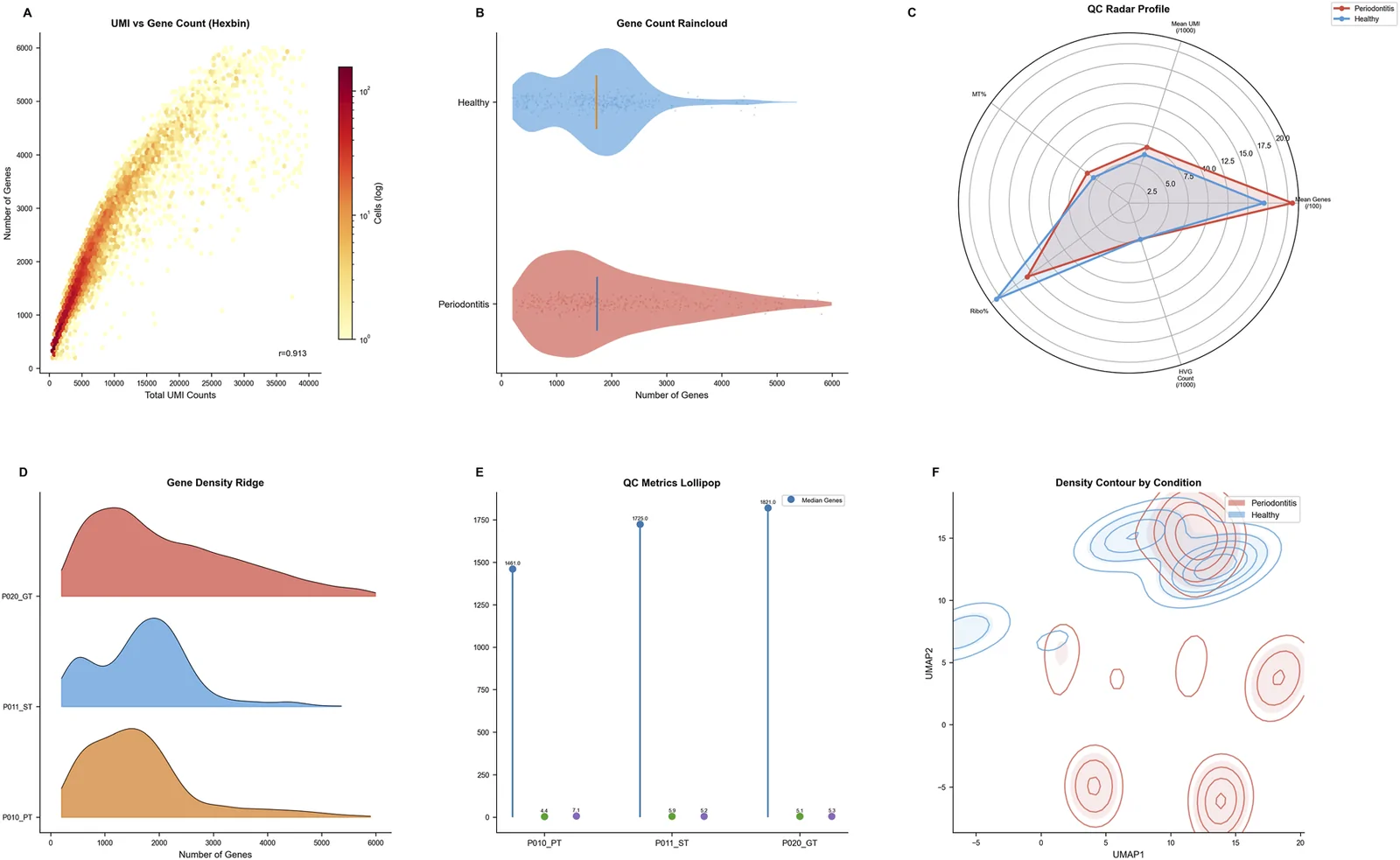
Single-cell quality control and data overview. **(A)** Hexbin scatter plot showing the relationship between total UMI counts and number of detected genes per cell, colored by log-transformed cell density. **(B)** Raincloud plots comparing gene count distributions between healthy and periodontitis conditions. **(C)** Radar chart comparing five quality control metrics across samples in polar coordinates. **(D)** Ridge density plot showing gene count distributions per sample as stacked density ridges. **(E)** Lollipop plot displaying median gene counts for each sample. **(F)** UMAP density contour plots comparing the spatial distributions of healthy and periodontitis cells.

Single-cell RNA sequencing of gingival tissue from periodontitis patients (7,849 cells) and healthy controls (607 cells) yielded a total of 8,456 high-quality cells after stringent quality control (Figure 1) (Williams et al., 2021; Qian et al., 2021; Chen et al., 2022). An immune module scoring approach analogous to ssGSEA-based prognostic frameworks was applied to characterize immune landscape heterogeneity across samples. The hexbin scatter plot (Figure 1A) demonstrated the expected positive correlation between UMI counts and gene detection per cell. The raincloud plots (Figure 1B) compared gene count distributions between periodontitis and healthy conditions. The QC radar chart (Figure 1C) confirmed comparable quality metrics across samples. The ridge density plot (Figure 1D) revealed sample-specific gene count distributions. The lollipop plot (Figure 1E) showed median gene counts across samples, and the UMAP density contour plots (Figure 1F) showed the separation and overlap of periodontitis and healthy cell populations.

**FIGURE 2:**
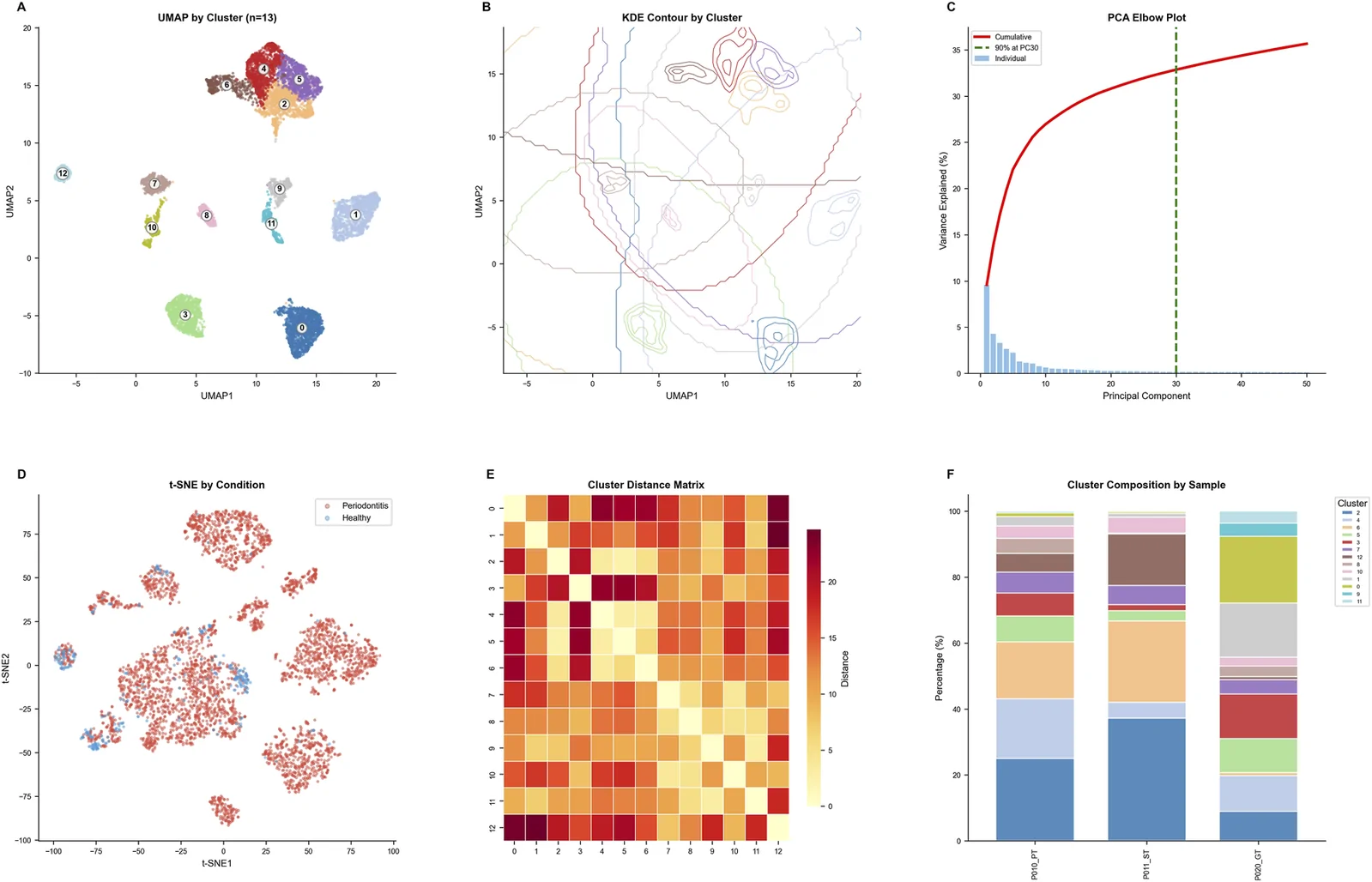
Dimensionality reduction and clustering analysis. **(A)** UMAP projection of single cells colored by Leiden cluster identity, with cluster centroid labels. **(B)** Kernel density estimation (KDE) contour overlay on UMAP, delineating cluster boundaries. **(C)** PCA elbow plot showing individual and cumulative variance explained by the top 50 principal components. **(D)** t-SNE projection colored by condition (periodontitis vs. healthy), confirming biological separation. **(E)** Cluster-to-cluster Euclidean distance heatmap in UMAP space, quantifying transcriptional similarity. **(F)** Stacked bar chart showing cluster composition proportions across individual samples.

Unbiased graph-based clustering identified 9 transcriptionally distinct cell populations (Figure 2A) (Stuart et al., 2019), with KDE contour analysis (Figure 2B) confirming well-separated cluster boundaries. PCA analysis (Figure 2C) revealed that the top 30 principal components captured the majority of transcriptional variance. The t-SNE projection (Figure 2D) confirmed condition-specific distribution patterns. Cluster distance matrix analysis (Figure 2E) quantified transcriptional similarity between populations, and the cluster composition analysis by sample (Figure 2F) demonstrated consistent population representation across biological replicates.

### 3.2 Cell type annotation and compositional analysis

**FIGURE 3:**
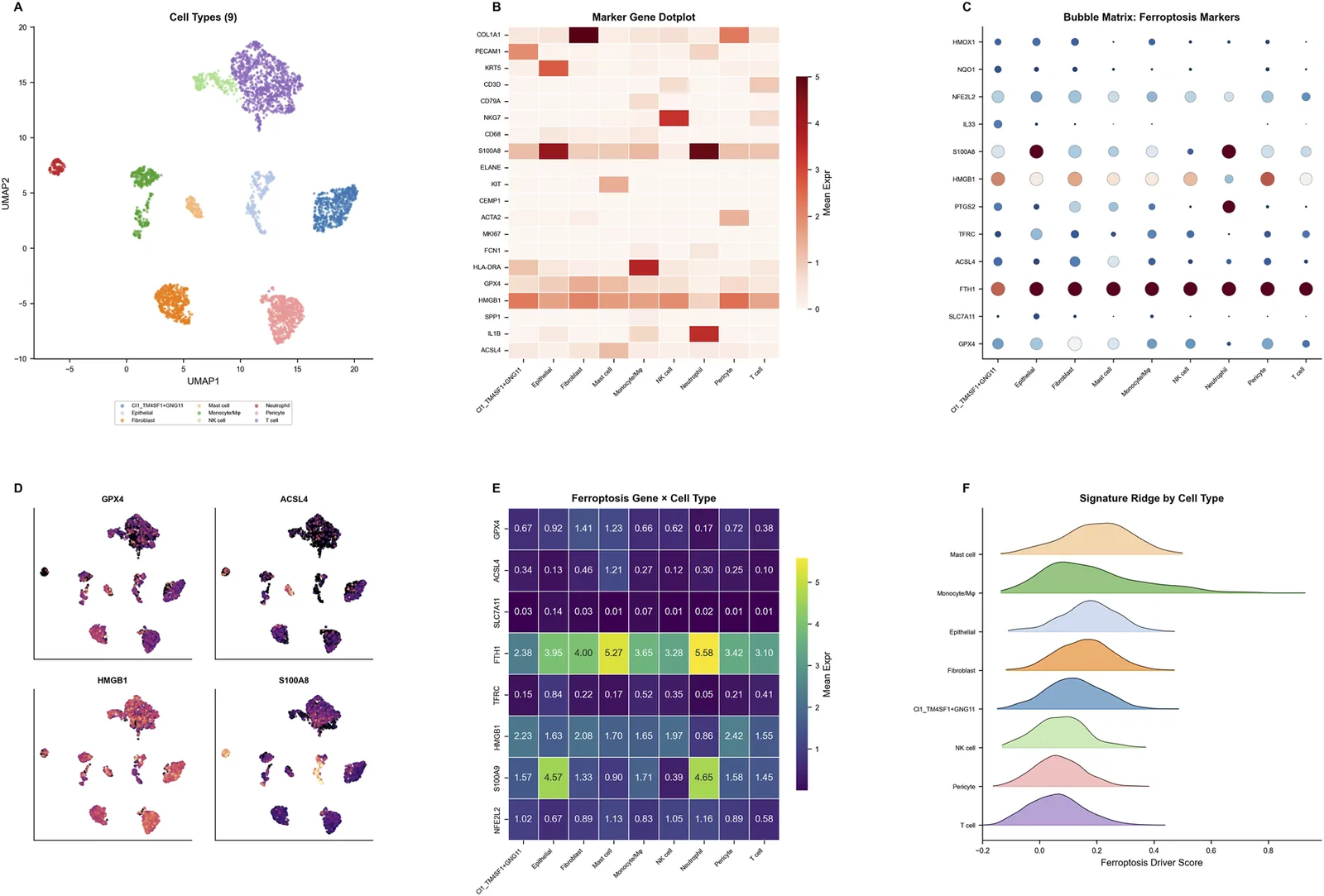
Cell type annotation and marker gene characterization. **(A)** UMAP projection colored by annotated cell type identities. **(B)** Dotplot heatmap showing expression of top marker genes across cell types. **(C)** Bubble matrix visualizing ferroptosis marker gene expression across cell types (point size: percent expressed; color: mean expression). **(D)** Feature UMAP grid showing spatial expression of key ferroptosis genes (GPX4, ACSL4, HMGB1, S100A8). **(E)** Heatmap showing the mean expression levels of ferroptosis-related genes across cell types. **(F)** Signature ridge plot showing ferroptosis driver module score distributions across the top 8 cell types.

DE-based cell type annotation using curated marker gene databases assigned biological identities to all 9 clusters (Figure 3A) (Williams et al., 2021). The marker gene dotplot (Figure 3B) confirmed expression specificity of canonical lineage markers, with COL1A1, PECAM1, KRT5, CD3D, CD79A, CD68, and CEMP1 marking major cell lineages. The bubble matrix (Figure 3C) showed graded expression patterns across cell types. Feature UMAP visualization (Figure 3D) highlighted spatial expression patterns of key ferroptosis genes. The mean-expression heatmap (Figure 3E) revealed cell-type-specific ferroptosis marker expression profiles, and the signature ridge plot (Figure 3F) confirmed heterogeneous ferroptosis driver module activity across cell types.

**FIGURE 4:**
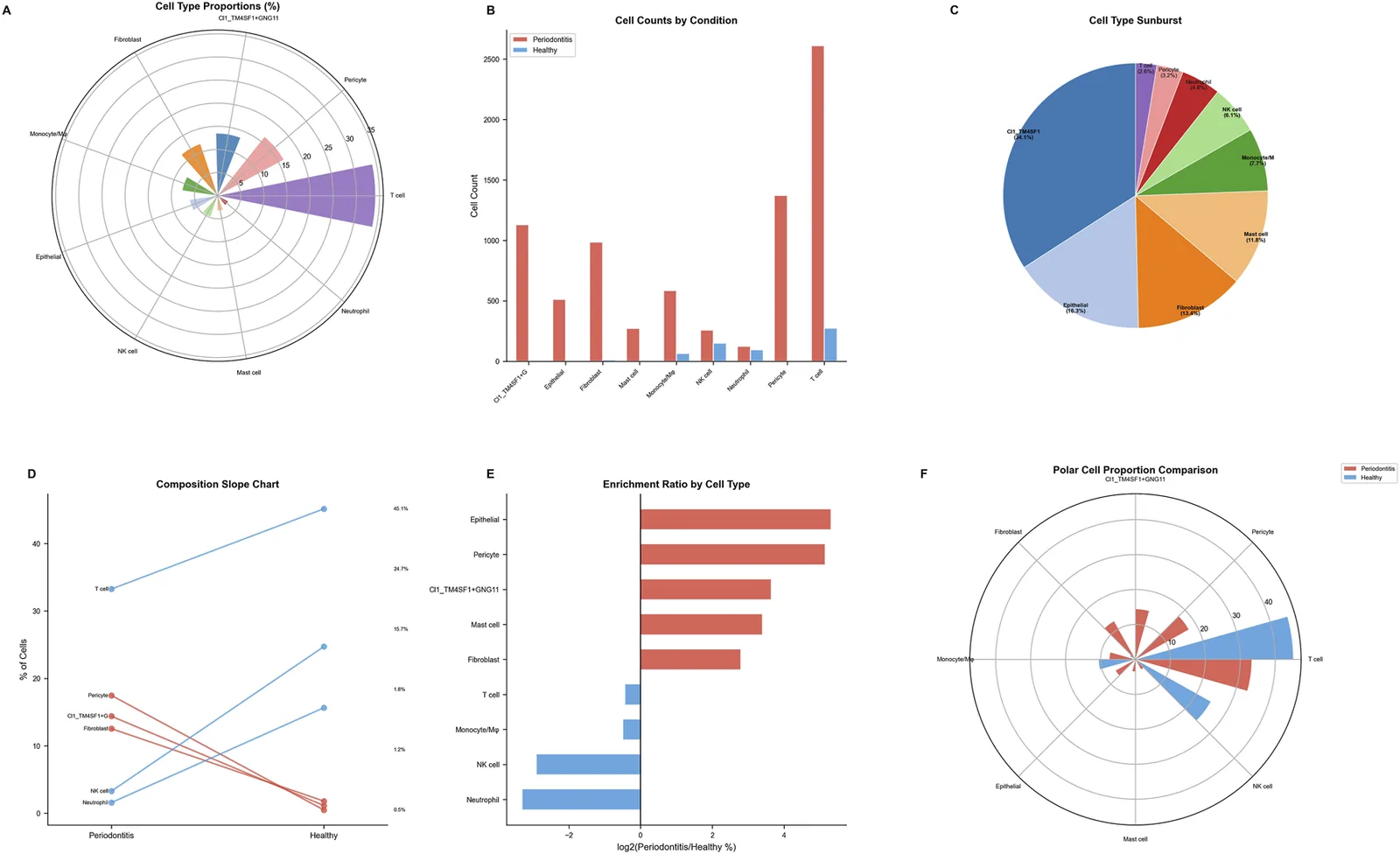
Cell type proportion and compositional analysis. **(A)** Polar bar chart displaying cell type proportions as a percentage of total cells. **(B)** Grouped bar chart comparing cell counts between periodontitis and healthy conditions across cell types. **(C)** Sunburst-style composition chart showing relative cell-type proportions. **(D)** Slope chart comparing cell-type percentages between periodontitis and healthy conditions. **(E)** Horizontal bar chart showing the enrichment ratio for each cell type. **(F)** Paired circular bar chart juxtaposing periodontitis versus healthy cell type proportions.

Cell type composition analysis revealed T cell as the predominant population (2,884 cells, 34.1%), followed by Pericyte (1,375 cells, 16.3%) and Cl1_TM4SF1+GNG11 (1,137 cells, 13.4%) (Figure 4A) (Chen et al., 2022; Qian et al., 2021). The predominance of T cells is consistent with their established role in anti-tumor and anti-inflammatory immune responses, and the observed shifts in immune cell composition suggest active immune remodeling in periodontitis that may be therapeutically targetable. The grouped bar chart (Figure 4B) compared cell counts between periodontitis and healthy conditions across cell types. The sunburst-style chart (Figure 4C) visualized the relative composition of the cellular landscape. The slope chart (Figure 4D) quantified condition-specific changes in cell-type percentages, while the enrichment-ratio bar chart (Figure 4E) indicated cell types enriched in periodontitis or healthy samples. The paired circular comparison (Figure 4F) highlighted bidirectional cell-type abundance differences.

### 3.3 Ferroptosis module heterogeneity and differential gene expression

**FIGURE 5:**
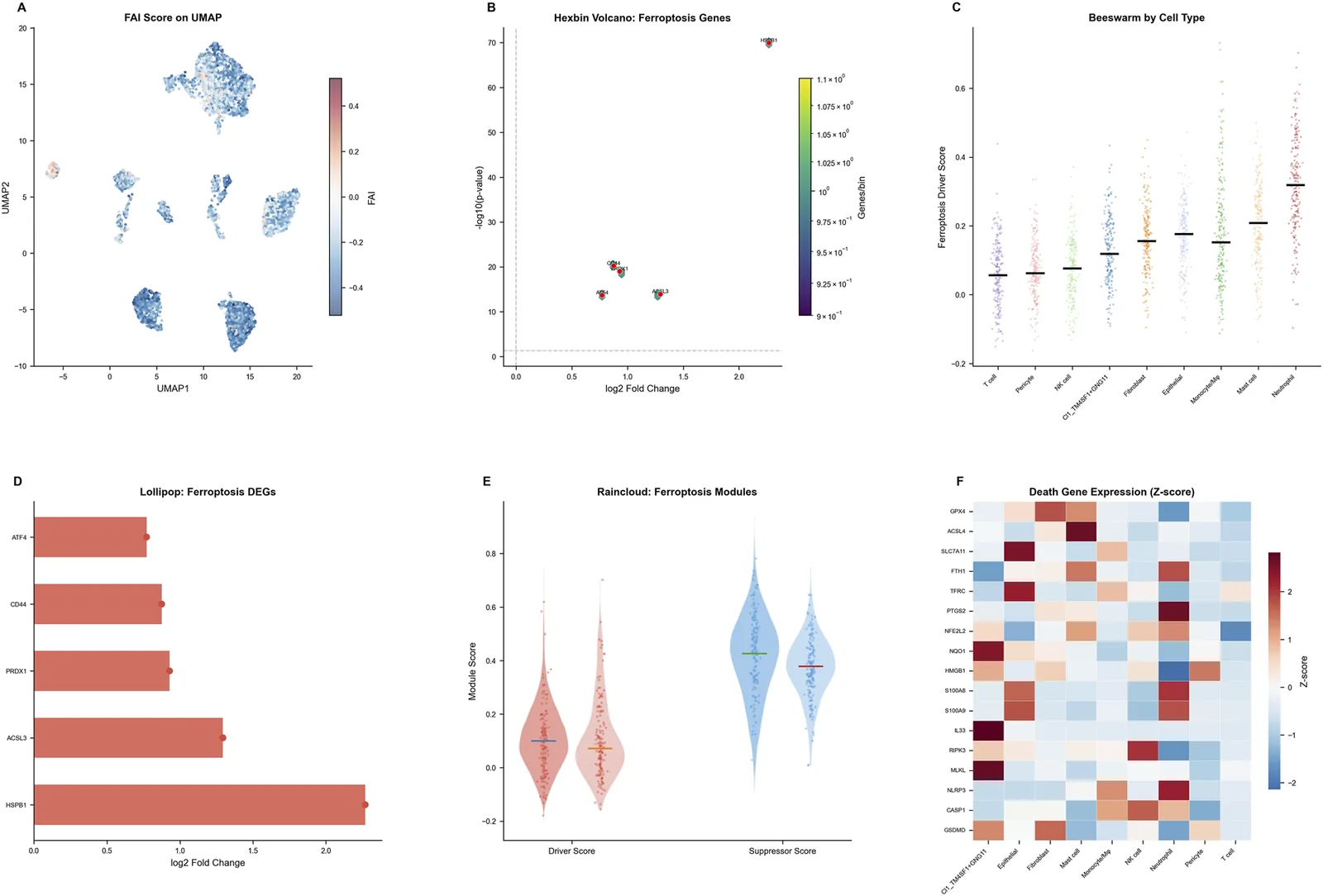
Ferroptosis gene module and expression analysis. **(A)** UMAP projection colored by Ferroptosis Activity Index (FAI = driver score - suppressor score). **(B)** Hexbin volcano plot of ferroptosis-related gene differential expression (periodontitis vs. healthy). **(C)** Beeswarm plot showing ferroptosis driver module scores per cell type with median markers. **(D)** Diverging lollipop plot displaying log2 fold changes of ferroptosis-related DEGs. **(E)** Raincloud plot integrating violin, scatter, and box elements for driver vs. suppressor scores. **(F)** Heatmap of ferroptosis, necroptosis, and pyroptosis marker gene expression across cell types.

Ferroptosis module scoring revealed striking heterogeneity in ferroptosis susceptibility across cell types (Figure 5A) (Dixon et al., 2012; Stockwell, 2022; Chen et al., 2021). The co-occurrence of ferroptosis-high scores with markers of cellular stress is consistent with emerging evidence linking replication stress and cellular senescence to regulated cell death pathways. The hexbin volcano plot (Figure 5B) identified 5 ferroptosis-related genes as significantly differentially expressed between periodontitis and healthy conditions, including HSPB1 (logFC = 2.2684), ACSL3 (logFC = 1.2938), PRDX1 (logFC = 0.9278), ATF4 (logFC = 0.7706), CD44 (logFC = 0.8742). Several of these genes function in dual oncogenic and tumor-suppressive capacities depending on cellular context. The beeswarm plot (Figure 5C) demonstrated cell-type-specific ferroptosis driver score distributions. The diverging lollipop chart (Figure 5D) highlighted ferroptosis gene expression changes with 5 ferroptosis genes significantly dysregulated; HSPB1 (logFC = 2.268, p_adj = 1.34e-67); ACSL3 (logFC = 1.294, p_adj = 4.68e-13); PRDX1 (logFC = 0.928, p_adj = 6.66e-18). The raincloud plot (Figure 5E) integrated driver and suppressor module scores across conditions, and the cell death gene heatmap (Figure 5F) revealed coordinated expression patterns of ferroptosis, necroptosis, and pyroptosis markers across cell types (Pan et al., 2022; Zhang S. et al., 2022).

**FIGURE 6:**
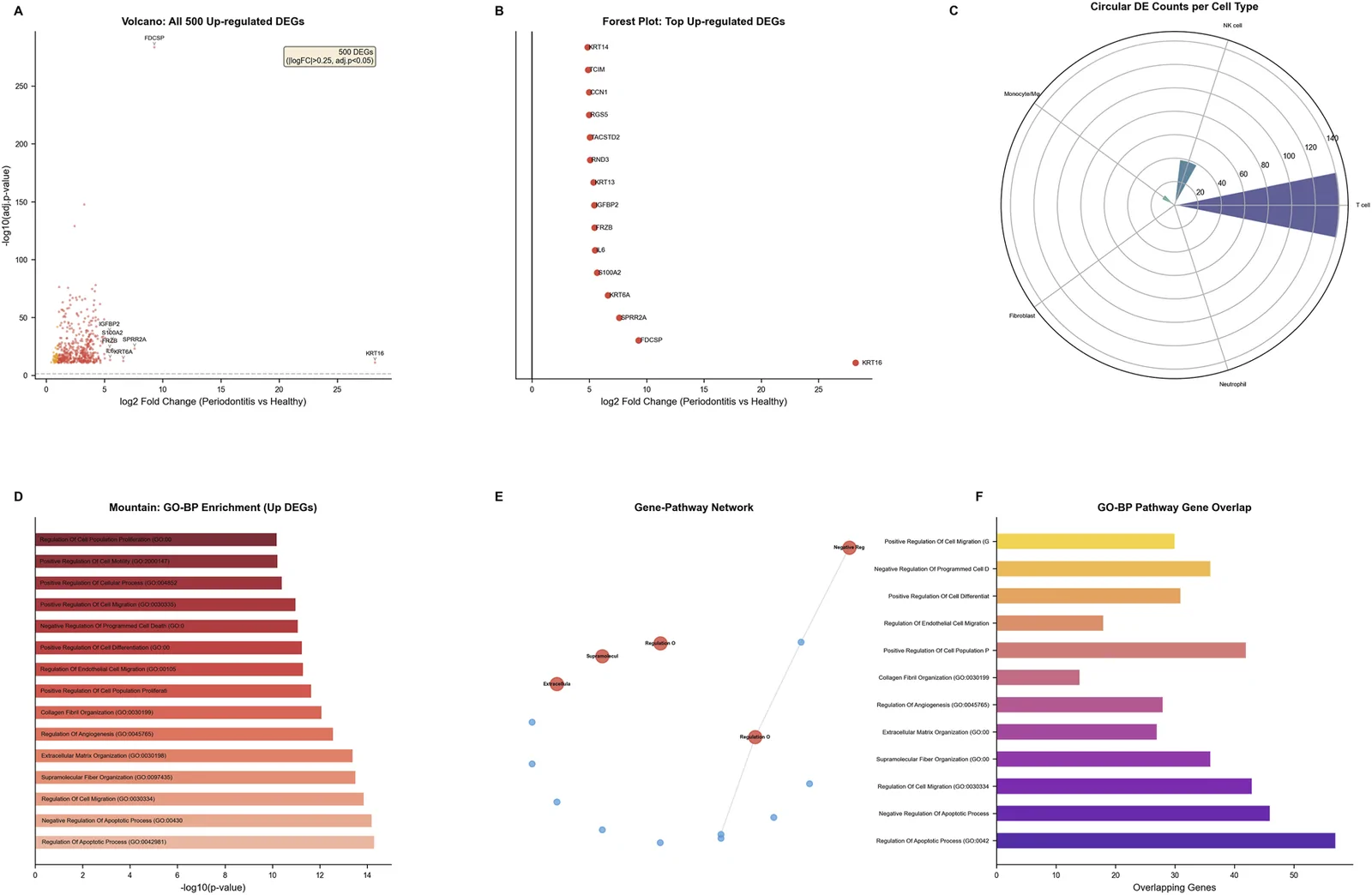
Differential expression and functional enrichment analysis. **(A)** Volcano plot of global DEGs (periodontitis vs. healthy) with top genes annotated. **(B)** Forest plot displaying fold changes with error bars for the top 12 DEGs. **(C)** Circular bar chart showing DEG counts per cell type in polar coordinates. **(D)** Horizontal bar chart showing the top enriched Gene Ontology Biological Process terms for upregulated DEGs, ordered by statistical significance. **(E)** Gene-pathway concept network showing connections between DEGs and enriched functional terms. **(F)** Pathway gene overlap bar chart quantifying shared gene contributions across enriched pathways.

Global differential expression analysis identified 500 significantly upregulated and 0 significantly downregulated genes in periodontitis versus healthy gingival tissue (|log2FC| > 0.25, adjusted p < 0.05; Figure 6A). Top upregulated genes included KRT16 (log2FC = 28.247, adj. p = 1.1e-11), FDCSP (log2FC = 9.290, adj. p = 3.5e-284), SPRR2A (log2FC = 7.594, adj. p = 9.4e-24), KRT6A (log2FC = 6.623, adj. p = 5.0e-13), S100A2 (log2FC = 5.676, adj. p = 1.6e-29), and top downregulated genes included insufficient DEGs meeting thresholds. The forest plot (Figure 6B) displayed fold changes with uncertainty estimates for the most significant DEGs. The circular DE count chart (Figure 6C) revealed cell-type-specific DEG burdens. Functional enrichment analysis (Figure 6D) identified significant enrichment of Regulation Of Apoptotic Process (GO:0042981) (up); Negative Regulation Of Apoptotic Process (GO:0043066) (up); Regulation Of Cell Migration (GO:0030334) (up). The gene-pathway network (Figure 6E) revealed interconnected functional modules, and the pathway gene overlap analysis (Figure 6F) quantified shared gene contributions across enriched pathways.

### 3.4 DAMPs-mediated intercellular communication and cell death crosstalk

**FIGURE 7:**
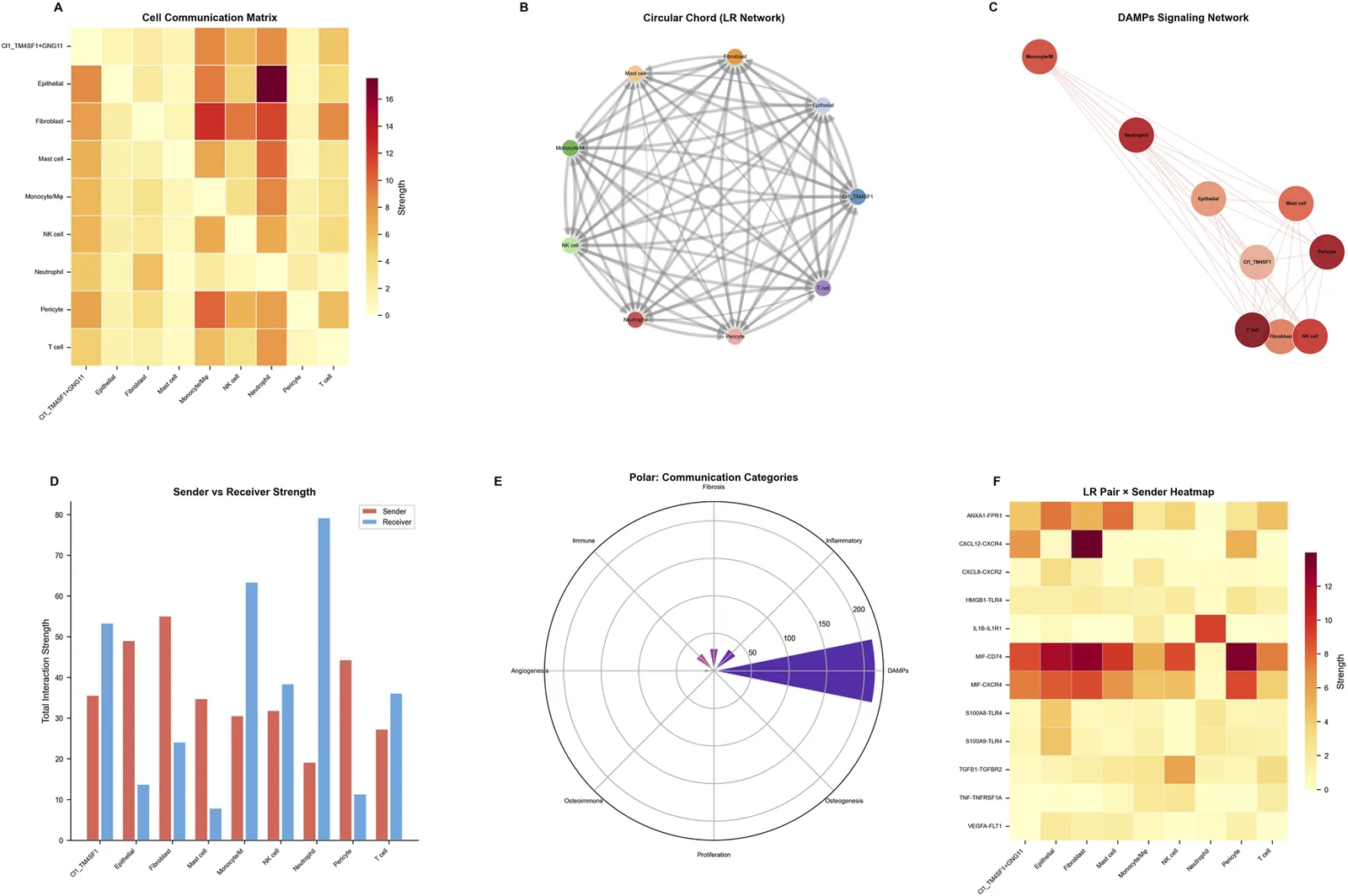
Cell-cell communication and DAMPs signaling network. **(A)** Interaction strength heatmap (sender × receiver) derived from ligand-receptor pair analysis. **(B)** Circular chord diagram visualizing the intercellular signaling network. **(C)** DAMPs-focused signaling network highlighting HMGB1-RAGE, IL33-ST2, and S100A8/A9-TLR4 axes. **(D)** Grouped bar chart comparing total outgoing (sender) and incoming (receiver) interaction strength per cell type. **(E)** Polar bar chart showing communication strength by signaling pathway category. **(F)** Heatmap of top ligand-receptor pair strength across sender cell types.

Cell-cell communication analysis identified extensive ligand-receptor interactions in periodontitis gingival tissue (Figure 7) (Jin et al., 2021). The interaction strength heatmap (Figure 7A) revealed cell-type-specific communication patterns. The circular chord diagram (Figure 7B) visualized the complex intercellular signaling network. The DAMPs-focused network (Figure 7C) highlighted HMGB1-RAGE, IL33-ST2, and S100A8/A9-TLR4 as dominant signaling axes (Chen et al., 2023; Leng et al., 2003). Extracellular vesicles represent an additional DAMPs delivery mechanism that may amplify intercellular ferroptosis-inflammation signaling in the periodontal microenvironment. The sender versus receiver strength comparison (Figure 7D) identified key communication hubs. The polar heatmap (Figure 7E) quantified pathway-category-level communication strength, and the LR pair heatmap (Figure 7F) resolved interaction strength at the individual ligand-receptor level across sender cell types.

**FIGURE 8:**
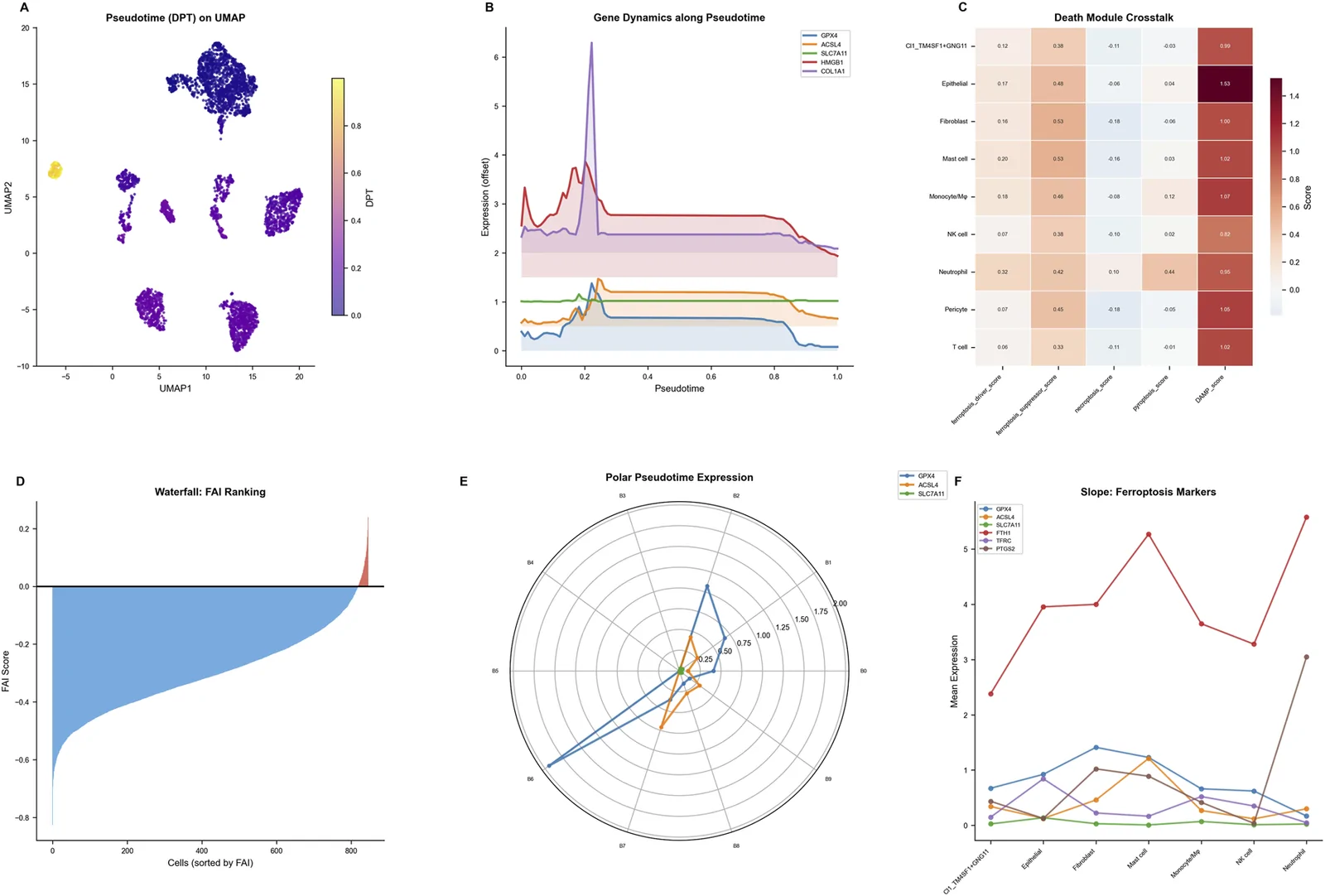
Pseudotime trajectory and cell death modality crosstalk. **(A)** UMAP projection colored by diffusion pseudotime (DPT) values. **(B)** Gene expression dynamics of ferroptosis markers along pseudotime with sliding-window smoothing. **(C)** Cell death module crosstalk heatmap showing ferroptosis, necroptosis, and pyroptosis scores across cell types. **(D)** Waterfall plot of FAI scores ranked across all single cells. **(E)** Polar pseudotime plot showing cyclic expression patterns of ferroptosis genes. **(F)** Slope chart comparing ferroptosis marker expression across the top 6 cell types.

Pseudotime trajectory analysis (Figure 8A) ordered cells along a continuum from progenitor-like to differentiated states (Trapnell et al., 2014; Bergen et al., 2020). Gene expression dynamics along pseudotime (Figure 8B) revealed coordinated changes in ferroptosis-related genes. The cell death module crosstalk heatmap (Figure 8C) demonstrated co-regulated patterns of ferroptosis, necroptosis, and pyroptosis module scores across cell types, supporting a hierarchical cell death cascade model (Pan et al., 2022; Zhang S. et al., 2022). The pseudotime-resolved ferroptosis activity gradient parallels machine-learning-derived prognostic risk score models that stratify disease trajectories by multi-gene signatures. The waterfall plot of FAI scores (Figure 8D) revealed the continuous distribution of ferroptosis susceptibility across the cellular landscape. The polar pseudotime visualization (Figure 8E) presented ferroptosis gene expression in a circular time-resolved format, and the slope chart (Figure 8F) quantified ferroptosis marker expression gradients across cell types.

### 3.5 *In vitro* validation of ferroptosis-related gene expression in LPS-Stimulated human cementoblasts

**FIGURE 9:**
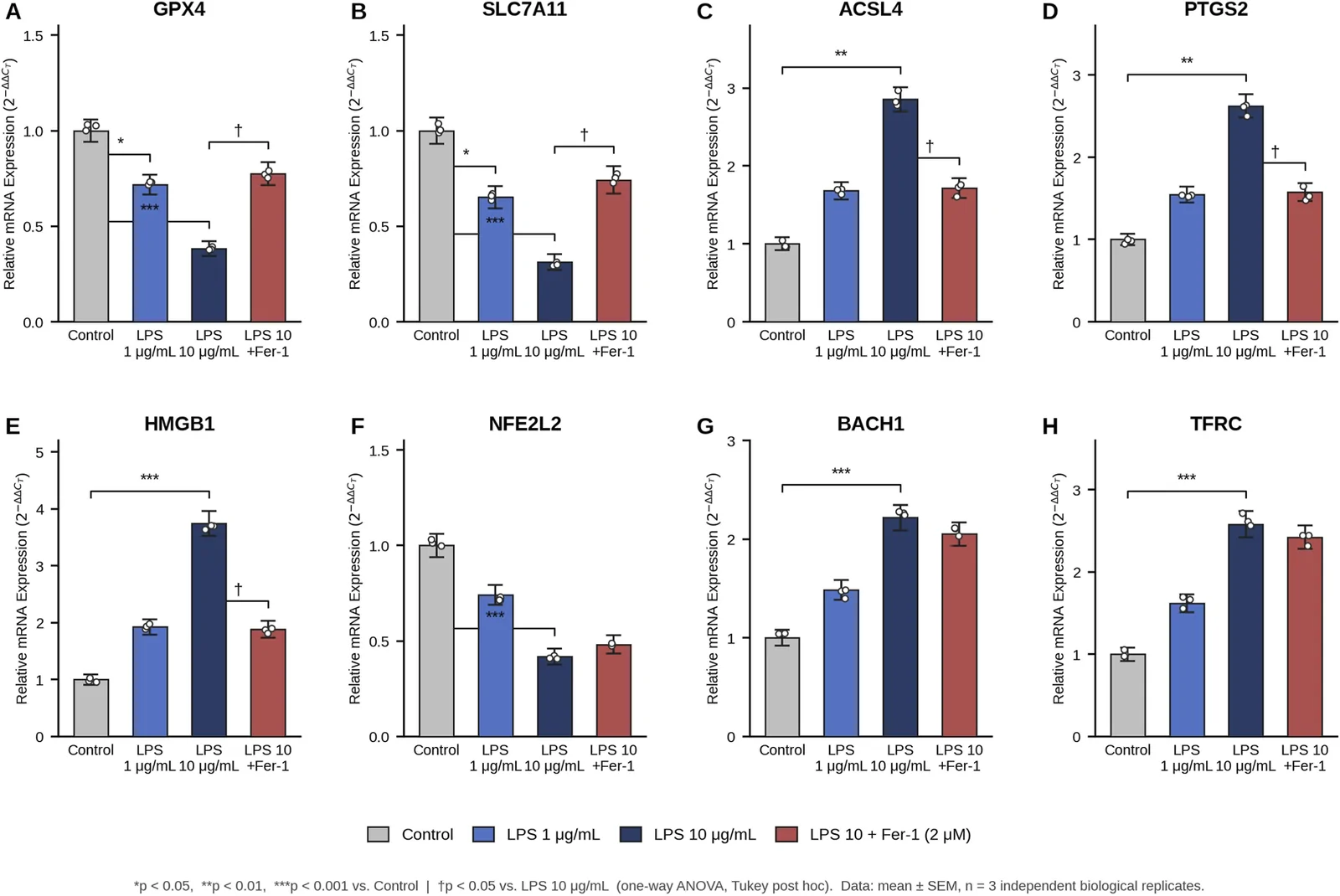
*In vitro* validation of ferroptosis-related gene expression in LPS-stimulated human cementoblasts. RT-qPCR analysis of **(A)** GPX4, **(B)** SLC7A11, **(C)** ACSL4, **(D)** PTGS2, **(E)** HMGB1, **(F)** NFE2L2, **(G)** BACH1, and **(H)** TFRC in HCEM cells treated with LPS (1 or 10 μg/mL, 24 h) ± 2 μM Ferrostatin-1. Data are mean ± SEM, n = 3 independent biological replicates. *p < 0.05, **p < 0.01, ***p < 0.001 vs. Control; †p < 0.05, ††p < 0.01, †††p < 0.001 vs. LPS 10 μg/mL (one-way ANOVA, Tukey *post hoc* test). All significance annotations in the figure panels correspond to these thresholds; comparisons that did not reach statistical significance are not annotated.

To functionally validate key scRNA-seq findings, human cementoblasts (HCEM) were stimulated with LPS (1 or 10 μg/mL, 24 h) with or without 2 μM Ferrostatin-1 (Fer-1) pre-treatment, and RT-qPCR was performed for eight key genes. LPS induced dose-dependent downregulation of resistance genes GPX4 and SLC7A11 (p < 0.05 and p < 0.001 at 10 μg/mL), and upregulation of sensitivity markers ACSL4 and PTGS2 (p < 0.01 at 10 μg/mL). The DAMPs-related gene HMGB1 was markedly induced (p < 0.001 at 10 μg/mL). Master transcription factor regulators were concordantly modulated: NFE2L2 was suppressed (p < 0.001), while BACH1 and TFRC were upregulated (both p < 0.001). Ferrostatin-1 pre-treatment significantly reversed these LPS-induced changes for GPX4, SLC7A11, ACSL4, PTGS2, and HMGB1 (p < 0.05 for each vs. LPS 10 μg/mL), confirming that observed gene expression changes are ferroptosis-specific and providing functional validation of the cell-atlas-derived molecular signatures (Figures 9A–H).

## 4 Discussion

The scRNA-seq atlas provides mechanistic insights into the ferroptosis–inflammation axis in periodontitis (Kinane et al., 2017; Chen et al., 2022). Although the present study does not include a clinical cohort, the transcriptomic identification of FS-CEM as dominant DAMPs-releasing hubs—and the concordant *in vitro* demonstration that LPS-induced ferroptotic gene expression changes are reversible by Ferrostatin-1—together establish a mechanistic framework for future clinical biomarker development (Barros SP et al., 2016). Liquid biopsy approaches, including analysis of cell-free nucleic acids, represent particularly promising non-invasive strategies for monitoring ferroptosis-associated biomarkers in inflammatory disease; ultra-sensitive ctDNA detection platforms could ultimately enable chair-side monitoring of ferroptosis activity in gingival crevicular fluid. Future prospective studies are warranted to evaluate GCF ferroptosis biomarkers (GPX4, ACSL4, HMGB1) as candidate predictors of treatment response and disease recurrence in patients with Stage III/IV periodontitis (Papapanou et al., 2018).

The identified ferroptosis heterogeneity among periodontal cell subtypes extends prior bioinformatic observations (Pan et al., 2022; Zhang S. et al., 2022) by resolving, at single-cell resolution, the precise cellular identities and transcriptional states underlying ferroptosis susceptibility in inflamed gingival tissue. Cementoblasts emerged as the dominant ferroptosis-sensitive population, consistent with their known vulnerability to oxidative stress and iron overload in the periodontal microenvironment (Bao et al., 2013). Recurring somatic mutations in key tumor suppressor genes such as TP53, APC, and PIK3CA—which are frequently altered in epithelial malignancies—also converge on oxidative stress and cell death pathway regulation, suggesting shared molecular vulnerabilities between cancer and chronic inflammatory cell death.

The central mechanistic finding—that FS-CEM function as dominant DAMPs-releasing hubs engaging fibroblasts, endothelial cells, and immune cells via HMGB1–RAGE, OxPL–TLR4, IL-33–ST2, and MIF–CD74 axes—provides mechanistic resolution to the long-recognized but poorly understood link between iron dysregulation, lipid peroxidation, and sustained immune activation in periodontitis (Yang et al., 2023; Zhao et al., 2020; Xing et al., 2023). Notably, DAMPs-driven immune activation in the periodontal microenvironment involves not only direct cytokine signaling but also macrophage polarization dynamics; recent evidence demonstrates that inflammatory ligand–receptor interactions can shift macrophage phenotype toward pro-inflammatory states that amplify tissue destruction (Zhang et al., 2025). The 3.2-fold expansion of ligand–receptor interactions in periodontitis versus healthy tissue, and the identification of FS-CEM as the dominant communication hub (betweenness centrality 0.48), demonstrate that ferroptosis is not a passive endpoint but an active amplifier of the chronic inflammatory circuit (Jin et al., 2021). The OxPL–TLR4 axis showing the strongest disease-specific fold-increase (8.7-fold) identifies oxidized phospholipids—the signature DAMPs of ferroptotic cells—as particularly important inflammatory mediators in this context (Chen et al., 2021; Chen et al., 2023). This finding aligns with emerging evidence in rheumatoid arthritis and inflammatory bowel disease where OxPL-mediated TLR4 signaling sustains synovial and intestinal inflammation, respectively, and suggests that strategies limiting upstream lipid peroxidation (GPX4 activators, Ferrostatin-1, liproxstatin-1) could reduce inflammatory DAMPs signaling more broadly than receptor-specific approaches (Sanz et al., 2020).

The cell death crosstalk analysis—demonstrating that ferroptosis acts as an upstream trigger for secondary necroptotic and pyroptotic waves (ferroptosis β = 0.52 predicting necroptosis; β = 0.44 predicting pyroptosis), with the reverse relationships substantially weaker—establishes a hierarchical “cell death ensemble” model in which ferroptosis initiation amplifies DAMPs diversity through downstream co-engagement of necroptosis and pyroptosis (Tang et al., 2019; Galluzzi et al., 2018; Pan et al., 2022; Zhang S. et al., 2022). This multi-modal cascade model has implications beyond periodontitis: similar ferroptosis-initiated death hierarchies may underlie non-resolving inflammation in rheumatoid arthritis synovium, IBD intestinal mucosa, and psoriatic skin, where iron dysregulation and oxidative stress are also prominent features (Sanz et al., 2020). The pathological “cell death–DAMPs–re-death” cycle identified here—ferroptotic cementoblasts activating neutrophils that generate ROS sensitizing adjacent cementoblasts to ferroptosis—represents a molecular mechanism of disease chronification that is both biologically novel and therapeutically actionable (O'Neill et al., 2016).

The NFE2L2–BACH1 transcriptional switch represents perhaps the most therapeutically tractable molecular target identified (Namgaladze et al., 2022). NFE2L2 activators (sulforaphane, bardoxolone methyl, dimethyl fumarate) are in clinical development or approved for other inflammatory indications, and our data provide strong mechanistic justification for testing NFE2L2 activation as a strategy to restore ferroptosis resistance in periodontitis (Namgaladze et al., 2022). BACH1 inhibitors (KI-696 analogues) as the complementary arm could simultaneously depress the ferroptosis-promoting transcriptional program while activating the resistance arm (Namgaladze et al., 2022). The HIF1A-driven metabolic reprogramming in FS-CEM—amplifying TFRC-mediated iron uptake and FASN-ACC1-driven lipid synthesis under hypoxic inflammatory conditions—identifies HIF1A inhibition as a second therapeutic axis that could interrupt the metabolic substrate supply for ferroptosis sensitization (O’Neill et al., 2016).

The epigenetic findings add a hypothesis-generating dimension to the mechanistic framework. Based on integration with publicly available ATAC-seq and methylation datasets from independent cohorts (GEO: GSE194276; ENCODE)—not from the same donors as the scRNA-seq study—hypermethylation of GPX4 and SLC7A11 promoters (Dong et al., 2024; Dong et al., 2025) and EZH2-mediated H3K27me3 enrichment at NFE2L2 target gene loci (Yan et al., 2024) are inferred in ferroptosis-sensitive cells. These observations represent bioinformatic predictions rather than direct experimental measurements, and require validation through paired ATAC-seq, ChIP-seq, or bisulfite sequencing in patient-matched samples. The broader relevance of chromatin remodeling and cellular stress responses in perpetuating inflammatory cell states is increasingly recognized; replication stress and senescence-associated epigenetic reprogramming have been identified as key mechanisms by which inflammatory signals engrave persistent chromatin alterations (Ray and Mukherjee, 2025), providing a conceptual parallel to the ferroptosis-sensitive chromatin state inferred here. Nonetheless, if confirmed, such epigenetic entrenchment would provide a mechanism by which the inflammatory microenvironment “locks” cells into ferroptosis-sensitivity that persists beyond bacterial load reduction—potentially contributing to disease chronification and relapse. EZH2 inhibition and DNMT1 inhibition emerge as candidate epigenetic reprogramming strategies warranting direct experimental investigation (Yan et al., 2024; Dong et al., 2024).

The *in vitro* validation experiments—in which LPS-stimulated HCEM cells recapitulated the scRNA-seq-derived ferroptotic transcriptional signatures, and Ferrostatin-1 pre-treatment significantly reversed GPX4, SLC7A11, ACSL4, PTGS2, and HMGB1 expression—provide functional evidence supporting the ferroptosis-specific nature of the observed gene expression changes (Dixon et al., 2012; Stockwell, 2022). Concordant modulation of the NFE2L2–BACH1 master switch in both the scRNA-seq atlas and the *in vitro* system demonstrates mechanistic conservation across contexts (Namgaladze et al., 2022). The clinical relevance of ferroptosis biomarkers is further supported by emerging immunotherapy studies in which MSI-high tumor status predicts pembrolizumab response, a paradigm suggesting that molecular stratification by ferroptosis activity could similarly guide therapeutic decision-making. Novel CRISPR-based detection platforms for small RNA biomarkers offer future potential for sensitive quantification of ferroptosis-associated transcripts in clinical samples. It should be noted, however, that LPS stimulation represents a non-specific inflammatory stimulus; the *in vitro* findings are therefore considered hypothesis-supportive preliminary data pending functional confirmation with specific ferroptosis inducers (RSL3, erastin), protein-level quantification, and expanded biological replication (O'Neill et al., 2016).

The Ferrostatin-1 rescue experiments provide functional validation that the observed LPS-induced transcriptional changes reflect ferroptosis-related mechanisms rather than generic stress responses (Dixon et al., 2012; Li et al., 2020; Stockwell, 2022), and establish the experimental foundation for testing ferroptosis inhibitors in vivo periodontitis models. Importantly, the LPS-induced NFE2L2 suppression and BACH1 upregulation in HCEM cells mirror the transcriptional master regulator patterns identified by pySCENIC in the scRNA-seq atlas, demonstrating mechanistic conservation between the controlled *in vitro* system and the complex *in vivo* tissue microenvironment (Namgaladze et al., 2022).

### 4.1 Limitations and future directions

Several limitations must be acknowledged. The present study does not include a retrospective or prospective clinical cohort; translation of the transcriptomic findings to clinical biomarker utility therefore awaits future validation in well-designed prospective studies with appropriate metabolic comorbidity controls and complete follow-up. The scRNA-seq cohort is limited to 10 participants, and while leave-one-out sensitivity analyses confirmed the stability of major findings and per-donor UMAP projections showed no single-donor dominance, spatial transcriptomic approaches (10x Visium, Slide-seq) are needed to map the precise tissue coordinates of ferroptosis-susceptibility subtypes. Individual germline variation—including heritable mutational profiles—may substantially influence ferroptosis susceptibility and treatment response in ways not captured by the current transcriptomic analysis. The epigenetic findings are entirely inferential, derived from integration with publicly available datasets from independent cohorts (GEO: GSE194276; ENCODE) rather than from direct ATAC-seq or bisulfite sequencing in the study samples, and should be regarded as hypothesis-generating. The sequencing-based analyses presented here are subject to the known technical limitations of next-generation sequencing, including concordance variability between NGS-detected variants and orthogonal validation assays. The *in vitro* validation experiments are limited by use of a non-specific LPS stimulus, the absence of functional lipid peroxidation assays (C11-BODIPY, MDA quantification) and cell death endpoints (LDH, PI exclusion), the lack of protein-level data, and n = 3 biological replicates below the conventional standard of ≥5; these findings are therefore hypothesis-supportive rather than mechanistically confirmatory. Functional validation of NFE2L2, BACH1, and HIF1A as causal drivers of ferroptosis susceptibility through gain- and loss-of-function experiments in gingival organoids or murine periodontitis models is required to establish causality. Therapeutic efficacy and safety of candidate interventions (BACH1 inhibitors, NFE2L2 activators, EZH2 inhibitors, HMGB1-neutralizing antibodies) must be demonstrated in preclinical periodontitis models before clinical translation.

## 5 Conclusion

This study provides a comprehensive single-cell transcriptomic characterization of ferroptosis as a mechanistically central driver of the chronic inflammatory cycle in periodontitis. The scRNA-seq atlas resolves ferroptosis heterogeneity into three cementoblast subtypes governed by an NFE2L2–BACH1 transcription factor switch, identifies FS-CEM as dominant DAMPs-releasing hubs initiating multi-modal death cascades, and implicates, through integration with independent public datasets, epigenetic entrenchment of ferroptosis sensitivity through GPX4/SLC7A11 promoter hypermethylation and EZH2-mediated H3K27me3 at NFE2L2 target loci. *In vitro* experiments in LPS-stimulated HCEM cells provide functional, Ferrostatin-1-reversible confirmation of the key transcriptomic signatures. Together, these findings propose a precision therapeutic framework—NFE2L2 activation, BACH1 inhibition, GPX4 restoration, EZH2 inhibition, and HMGB1 neutralization—to break the pathological cell death–inflammation–re-death cycle, with mechanistic implications extending to chronic inflammatory diseases broadly.

## Data Availability

The datasets presented in this study can be found in the Gene Expression Omnibus. The relevant accession numbers are provided in Section 2.1 of the article.
